# Metabolome Consistency: Additional Parazoanthines from the Mediterranean Zoanthid *Parazoanthus* *Axinellae*

**DOI:** 10.3390/metabo4020421

**Published:** 2014-05-30

**Authors:** Coralie Audoin, Vincent Cocandeau, Olivier P. Thomas, Adrien Bruschini, Serge Holderith, Grégory Genta-Jouve

**Affiliations:** 1 Département Actifs, Direction Recherche & Technologie, Chanel Parfums Beauté, 8 rue du cheval blanc, 93500 Pantin, France; E-Mails: coralie.audoin@chanel-corp.com (C.A.); vincent.cocandeau@chanel-corp.com (V.C.); serge.holderith@chanel-corp.com (S.H.); 2 Nice Institute of Chemistry UMR 7272 CNRS-PCRE, University of Nice-Sophia Antipolis, Parc Valrose, 06108 Nice, France; E-Mail: olivier.thomas@unice.fr; 3 CTEL (Centre Transdisciplinaire d’Epistémologie de la Littérature et des Arts Vivants), EA 1758, Université Nice-Sophia Antipolis, 06103 Nice, France; E-Mail: bruschinihadrien@gmail.com; 4 Laboratoire de Pharmacognosie, UMR CNRS 8638 COMETE, Université Paris Descartes Sorbonne Paris Cité, 4 Avenue de l’Observatoire 75006 Paris, France

**Keywords:** LC-MS/MS, parazoanthine, zoanthid, *Parazoanthus axinellae*, ECD

## Abstract

Ultra-high pressure liquid chromatography coupled to high resolution mass spectrometry (UHPLC-MS/MS) analysis of the organic extract obtained from the Mediterranean zoanthid *Parazoanthus axinellae* yielded to the identification of five new parazoanthines F-J. The structures were fully determined by comparison of fragmentation patterns with those of previously isolated parazoathines and MS/MS spectra simulation of *in silico* predicted compounds according to the metabolome consistency. The absolute configuration of the new compounds has been assigned using on-line electronic circular dichroism (UHPLC-ECD). We thus demonstrated the potential of highly sensitive hyphenated techniques to characterize the structures of a whole family of natural products within the metabolome of a marine species. Minor compounds can be characterized using these techniques thus avoiding long isolation processes that may alter the structure of the natural products. These results are also of interest to identify putative bioactive compounds present at low concentration in a complex mixture.

## 1. Introduction

In our ongoing research on the characterization of the marine chemodiversity, we decided to extend and deepen knowledge of the chemical diversity produced by the Mediterranean zoanthid *Parazoanthus axinellae* (Schmidt, 1862) using a hyphenated approach. In our first chemical study, we described the isolation of five new hydantoin alkaloids named parazoanthines A–E (**1**–**5**) using a classic natural product chemistry approach involving extraction and purification followed by structure elucidation mainly based on 1D and 2D NMR data analyses [1]. Although similar approaches have already led to the isolation of thousands of natural products from a large diversity of living organisms [2], our knowledge of the full metabolome is limited by the sensitivity of the structure elucidation techniques, especially NMR. 

The identification of natural products from a known family with only minor structural modifications, such as chain elongation, methoxylation, halogenation should not require thorough purification to be perfectly described [3,4]. An important feature of such analytical development is based on the concept of metabolome consistency and the necessity for the analyst to decide whether or not a research for minor compounds can lead to new skeletons or just slight modifications of known scaffolds. The metabolome consistency can be described as the rules used by living organisms to produce the compounds often incorrectly named secondary metabolites. The rules are not only based on known chemical reactivity but also on selected biochemical pathways [5]. Prediction of potential metabolites based on chemical structures and biochemical pathways has been proposed earlier by Ridder *et al.* but has not been used for *de novo* identification of new natural products [6]. Using the knowledge of preliminary chemical studies, the natural products chemist can infer the possibility for the organism to modify a specific compound.

In this study, we decided to take advantage of the recent advances in the fields of metabolomics [7,8] and computer assisted structure elucidation to unambiguously identify five new compounds belonging to the parazoanthine family from the same zoanthid *P. axinellae*: parazoanthines F–J (**6**–**10**) together with previously described zoanthoxanthin like compounds [9] using modern ultra-high pressure liquid chromatography coupled to high resolution mass spectrometry (UHPLC-MS/MS) and logical inferences based on an established knowledge.

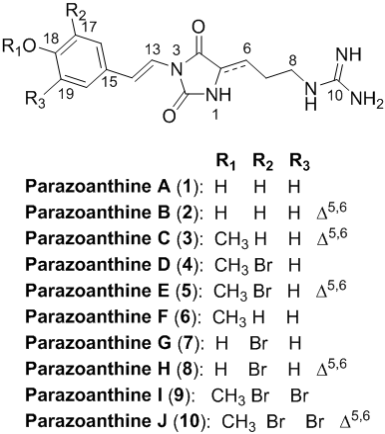


## 2. Results and Discussion

### 2.1. Description of the Approach

**Figure 1:**
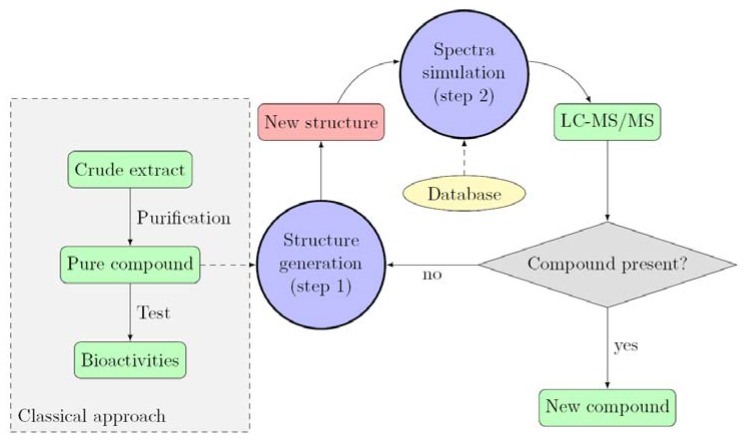
Proposed work-flow for the identification of new compounds.

The classical work-flow used for bioactive compounds discovery from natural origin is presented on Figure 1 (dashed line). The approach we propose to develop is based on the classical approach as it requires a fully characterized compound as an input for the generation of a new structure. This step of generation was performed using known chemical modifications observed in the studied organism (Database). From our previous study, several chemical transformations were observed such as oxidation (from **2** to **1**), bromination (from **3** to **5**) or methylation (from **2** to **3**). Based on this knowledge and the metabolome consistency, several other structures can be proposed as putative metabolites (step 1). The newly generated structures are then submitted to the step 2, consisting in the MS spectra generation of the putative compounds. In this step, spectra are generated using identified fragments from the previously identified compounds (in house database containing fragmentation information on the compounds class, in the present study, the parazoanthines). Looking at the parazoanthine structure, several modifications were considered. Several halogenations using bromine, chlorine, iodine and fluorine were implemented. Other substituents for the hydroxyl moiety were also used: methyl, ethyl and isopropyl.

Once the theoretical spectra have been simulated, they are compared with the experimental data obtained by UHPLC-MS/MS.

### 2.2. Identification of the Fragmentation Pattern

**Figure 2:**
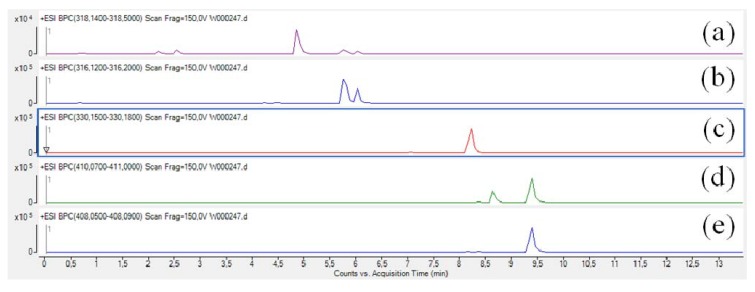
Extracted ions chromatograms (EIC) of the five known parazoanthines: (**a**) parazoanthine A, (**b**) parazoanthine B, (**c**) parazoanthine C, (**d**) parazoanthine D, (**e**) parazoanthine E.

After extraction with MeOH (5 mL), the crude extract was analyzed by UHPLC-MS/MS. We first focused on the five known parazoanthines A–E (**1**–**5**) in order to identify fragmentation patterns for this family of compounds and implement the database. As expected, all five target masses at *m*/*z* 318, 316, 330, 412 and 410 corresponding to parazoanthine A, B, C, D and E respectively were present in the extracted ions chromatograms (EIC) (Figure 2).

As depicted on Figure 2, two peaks are present on the EIC of parazoanthine D (**4**). The EIC on the pseudo-molecular ion of **4** at *m*/*z* 410 [C_16_H_20_^79^BrN_5_O_3_+H]^+^ also corresponds to the pseudo-molecular ion of compound **5** for the [C_16_H_18_^81^BrN_5_O_3_+H]^+^. In order to fully identify the different compounds **1**-**5**, the full scan acquisition was followed by three fragmentation scans using different collision energies (10 V, 20 V, 30 V) on the most intense ion. The obtained mass spectra were then used to confirm the structure of the compounds and established the fragmentation pattern.

**Figure 3:**
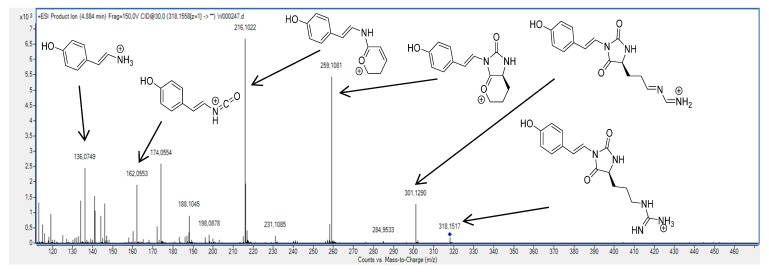
Fragmentation pattern of the parazoanthine A using a 20 V collision energy on the pseudo-molecular ion at *m*/*z* 318.1558.

The compounds belonging to the parazoanthine family are positively charged in acidic conditions due to the presence of a guanidine moiety (p*K*_a_ > 13) [10] and thus the acquisition was performed using a positive ionisation mode. As illustrated on Figure 3, several fragments were observed using a collision energy of 20 V on the pseudo-molecular ion at *m*/*z* 318.1558 (C_15_H_20_N_5_O_3_, ∆ 0.8 ppm) corresponding to parazoanthine A (**1**).

A first fragment at *m*/*z* 301.1290 (C_15_H_17_N_4_O_3_, ∆ 1.72 ppm) is clearly identified on the mass spectrum. This fragment is commonly observed in peptide for the guanidine moiety of arginine residues and results from the ammonia loss [11]. Another fragment, characteristic of the presence of a guanidine moiety, was observed at *m*/*z* 259.1081 (C_14_H_15_N_2_O_3_, ∆ –1.5 ppm) resulting from the neutral loss of CH_5_N_3_ by substitution. Although very abundant, these fragments are not very informative from a structural point of view and can be considered as markers of the guanidine alkaloids family. A third fragment at *m*/*z* 216.1012 (C_13_H_14_NO_2_, ∆ 3.3 ppm) corresponding to the loss of one isocyanic acid unit gives an indication on the presence of the hydantoin moiety. This fragmentation pattern was also observed for compounds **2**, **3**, **4** and **5**.

In summary, the fragmentation of compounds belonging to the parazoanthine family leads to five key fragments (up to six for several compounds). The identification method was applied to all known parazoanthines A–E and pseudo-molecular ions and fragments are presented in Table 1.

**Table 1:** Pseudo-molecular ions and characteristic fragments observed in ESI-(+) of compounds **1**–**5**.

Compound	Empirical Formula	*m*/*z* (∆ ppm)					
		[M+H]^+^	[M-NH_3_+H]^+^	[M-CH_5_N_3_+H]^+^	[M-C_2_H_6_N_4_O+H]^+^	[M-C_6_H_10_N_4_O+H]^+^	[M-C_7_H_10_N_4_O_2_+H]^+^
**1**	C_15_H_20_N_5_O_3_	318.1558 (0.8)	301.1290 (1.7)	259.1081 (-1.5)	216.1012 (3.3)	162.0553 (-2.1)	136.0749 (5.8)
**2**	C_15_H_18_N_5_O_3_	316.1410 (-1.8)	299.1135 (1.2)	257.0920 (0.3)	214.0867 (-2.1)	162.0551 (-0.9)	136.0725 (-6.2)
**3**	C_16_H_20_N_5_O_3_	330.1562 (-0.4)	313.1303 (-2.5)	271.1082 (-1.8)	228.1023 (-1.4)	176.0707 (-0.5)	150.0912 (-1.8)
**4**	C_16_H_20_BrN_5_O_3_	410.0829 (-1.6)	393.0555 (0.5)	351.0338 (0.2)	308.0277 (1.2)	253.9782 (1.2)	228.0022 (1.5)
**5**	C_16_H_18_BrN_5_O_3_	408.0676 (-2.5)	391.0406 (-1.5)	349.0192 (-2.8)	306.0115 (3.01)	253.9809 (0.9)	228.0031 (5.5)

For all known parazoanthines, the most probable calculated empirical formulae corresponded to the expected one and filtering of the generated empirical formula was also facilitated by the measurement of the relative isotopic abundance [12].

### 2.3. Identification of New Parazoanthines

**Figure 4:**
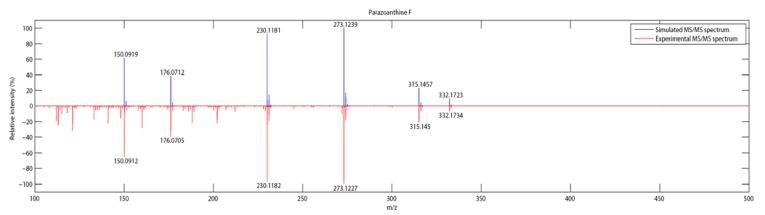
Comparison between simulated and experimental MS/MS spectra.

In order to identify additional parazoanthine derivatives in the extract, we first started the generation of new candidate structures. The first generation resulted in a compound already described in the literature as a *O*-methyl derivative of parazoanthine A (**1**) [13]. Generation of its fragmentation spectrum followed by a pattern matching gave a positive result as we observed a good agreement with the experimental spectra (Figure 4). The number of peaks observed on the simulated spectrum is reduced compared to the experimental one as we took into account only major fragmentation pathways (see supporting information) for the calculation of the daughter ion and neutral losses.

This result led to the conclusion that compound **6**, previously published by Manzo *et al.* and obtained by chemical synthesis [13], is present in the crude extract as a natural product. This result can be compared to tramadol, a common synthetic drug that has been isolated later from the root bark of *N. latifolia*, an African medicinal plant [14]. This approach has been used to generate several other compounds such as **7**, **8**, **9** and **10**. As for compound **6**, the pattern matching gave positive results and a good agreement was observed between simulated and experimental MS/MS spectra (Table 2).

**Table 2:** Pseudo-molecular ions and characteristic fragments observed in ESI-(+) of compounds **6**-**10**.

Compound	Empirical Formula	*m*/*z* (∆ ppm)					
		[M+H]^+^	[M-NH_3_+H]^+^	[M-CH_5_N_3_+H]^+^	[M-C_2_H_6_N_4_O+H]^+^	[M-C_6_H_10_N_4_O+H]^+^	[M-C_7_H_10_N_4_O_2_+H]^+^
**6**	C_16_H_21_N_5_O_3_	332.1734 (-5.1)	315.1450 (0.5)	273.1227 (2.5)	230.1182 (-2.8)	176.0705 (0.6)	150.0912 (-0.94)
**7**	C_15_H_18_BrN_5_O_3_	396.0611 (13.9)	379.0361 (10.4)	337.0158 (7.24)	294.0071 (-19.9)	-	-
**8**	C_15_H_16_BrN_5_O_3_	394.0496 (3.4)	377.0239 (1.3)	335.0011 (4.4)	291.9931 (12.6)	239.9640 (6.14)	-
**9**	C_16_H_19_Br_2_N_5_O_3_	487.9903 (5.0)	470.9640 (4.7)	438.9438 (1.4)	385,9386 (-0.1)	331.8931 (-4.7)	305.9108 (5.1)
**10**	C_16_H_17_Br_2_N_5_O_3_	485.9761 (2.0)	468.9540 (-7.4)	426.9300 (-2.9)	-	331.8917 (-0.2)	305.9025 (32.4)

**Figure 5:**
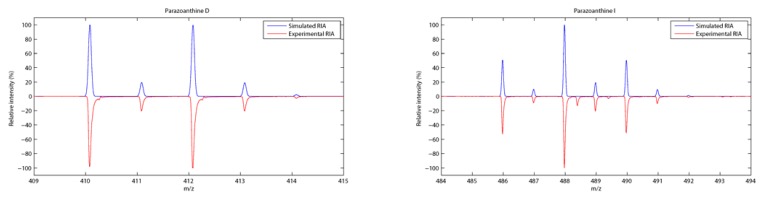
Relative isotopic abundance (RIA) of mono- and di-brominated compounds **4** (**left**) and **9** (**right**).

The pattern matching was even more interesting for the brominated compounds. The relative isotopic abundance (RIA) of bromine (due to the two most abundant isotopes ^79^Br and ^81^Br) indeed gave additional evidence for the compounds identification (Figure 5).

**Figure 6:**
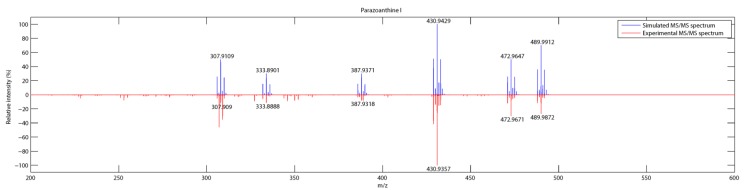
Comparison of simulated and experimental MS/MS spectra of parazoanthine I (9).

As depicted on Figure 6, the pattern matching using RIA gave an unambiguous identification of the fragments although the *m*/*z* selection window of 1 for the fragmentation leads to a loss in the accuracy of the measurement (**∆** > 5 ppm). For compound **9**, the main fragments are present but the relative abundance of the fragments is not as accurate as it was previously observed for non brominated compounds.

The present approach was rather efficient as five new compounds could be identified, while several other generated compounds could not be found in the extract. For instance, compounds **9** and **10** lacking the *O*-methyl substituent were not found as no spectral matching could be realized using the acquired data. This result can be interpreted as a chemical instability of the expected products. More probably it can be explained by the succession of the substitutive biochemical pathway. The absence of these compounds could be explained by a first bromination at the *ortho* position of the phenol followed by a methylation of the phenol and only in this case a second bromination could occur. Other *in silico* transformations did not lead to the identification of new compounds such as chlorinated, fluorinated or iodated compounds (see full list in supporting information).

It is important to highlight that after interpretation of the MS/MS data, some uncertainties remained but the use of metabolome consistency as a part of the identification process could overcome these issues. The position of the bromine is a good example on how the metabolome concistency can help. Looking at the phenol moiety, two positions are available for bromination, nonetheless, it is well known that one-electron oxidation of phenol gives a free radical from which unpaired electron can be delocalized via resonance forms favoring *ortho* and *para* positions. The use of the metabolome consistency must be used as a further evidence for the location of the bromine (in *ortho* position as previously observed for parazonathines D and E). It is also worth noting that out of 24186 compounds from the marine compounds database MarinLit (uptdate 23 February 2013), where reported compounds are identified using extended 1D and 2D NMR spectra, none of the brominated compounds presents a bromination in *meta* position only (see below).

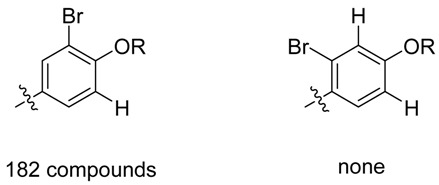


Some of the new compounds identified during this study contain one stereogenic center at C-5. As already published, the use of electronic circular dichroism enabled the determination of the absolute configuration for compounds **1** and **4** [1]. In the previous study, the acquisition of the ECD spectra was performed on the pure compounds but in the present case, we decided to turn to on-line detection in order to monitor the sign of the Cotton effect (CE) at 280 nm due to the minor amount of compounds present. This method has already been used for several natural products in the past, and proved highly efficient and sensitive [15,16,17].

### 2.4. Determination of the Absolute Configuration

**Figure 7:**
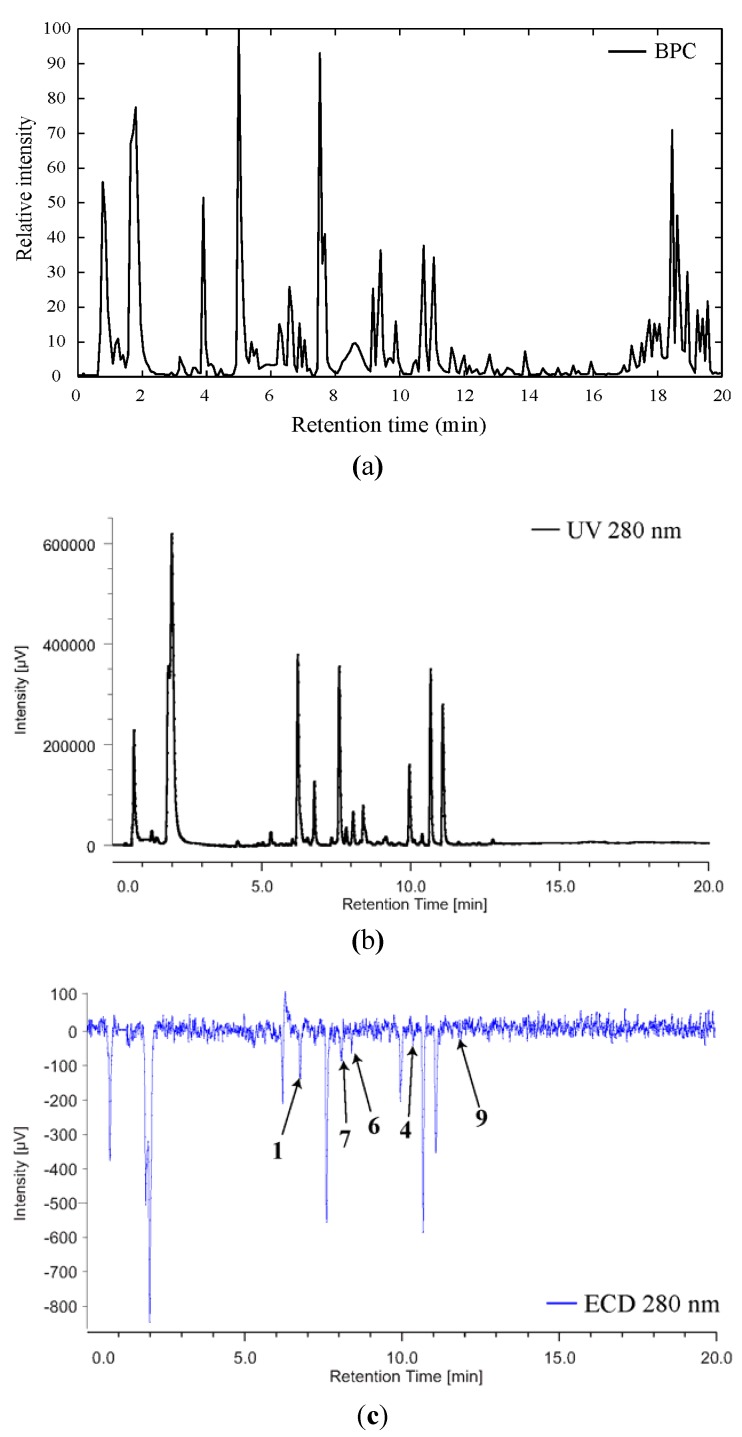
BPC (**a**), UV (**b**) and ECD (**c**) profiles of the crude extract of *Parazoanthus axinellae*.

In order to determine the absolute configuration of compounds **6**, **7** and **9**, we decided to use LC-ECD to measure the sign of the CE at 280 nm. Indeed, in our previous study we measured the maximum intensity of the CE at this wavelength [1]. As depicted on Figure 7, and although the signal is rather weak for some of the compounds, the sign of the CE at 280 nm is negative (must be of the opposite sign for the other enantiomer) for all the compounds of interest. It is worth noting that ECD measurement is performed at 280 nm which is specific for the parazoanthines chromophore, implying that the contribution to the CE at this wavelength of possible co-eluted compounds would be marginal.

This result was consistent with the one obtained for compounds **1** and **4** and the absolute configuration of compounds **6**, **7** and **9** could be assigned as *S*.

## 3. Experimental Section

### 3.1. General Procedure

UHPLC-ECD was performed with a 1200 Agilent system coupled with a Jasco XLC 3195CD detector. UHPLC-QTOF was conducted with a 1290 Agilent system coupled with an Accurate-Mass Q-TOF LC/MS 6520 (Agilent).

### 3.2. Biological Material

Colonies of *P. axinellae* (Schmidt, 1862) (Parazoanthidae) were collected as epibiont of the sponge *Cymbaxinella damicornis* by scuba (–30 m) off the coast of Villefranche-sur-Mer (“Grotte du Lido”) in January 2014.

### 3.3. Sample Preparation and Data Acquisition

The colonies of *P. axinellae* were carefully separated from the sponge *C. damicornis*. The fresh tissues were homogenised in 5 mL of MeOH using the Precellys homogeniser. For the chromatographic separation a C18 reversed-phase column (1.8 μm, 2.0 × 100 mm; Agilent) was used with a gradient of Water/Methanol/Formic acid/Ammonium formate 5 mM (from 95:5:0.1 to 5:95:0.1 in 15 min.). The mass spectral data was acquired in electrospray in positive mode. The gas heater, gas flow, nebulizer pressure were maintained at 300 °C, 10 and 40, respectively. Mass spectra were acquired over the *m*/*z* range of 100 to 1700 at a resolution of 12,000 FWHM (full width half at maximum) mass resolution. MS/MS spectra were acquired using three different collision energies as follow: 10, 20, 30 V on the three most intense ions of the full scan. UHPLC-DAD-ECD was performed using the same conditions as described for the UHPLC-MS in order to obtain comparable data.

### 3.4. MS/MS Spectra Simulation

Simulation of the MS/MS spectra was based on the observed fragments from the known compounds **1**–**5**. The fragmentation pattern was manually determined, the identification of the neutral losses was performed and the relative intensities of each fragment were measured and stored in a database. After the *in silico* generation of the new structures, the fragmentation pattern was applied and the mass of the fragments was calculated. The fragments intensities were generated randomly using the intensities values similar to those encountered for compounds **1**–**5**. A pattern matching of the calculated spectra was then performed using an automated approach, using a least square root regression on each scan of the LC-MS/MS chromatogram. Several spectra for each newly compounds were generated and compared to the experimental data in order to improve the intensity fitting. In the case of positive spectral matching, the most similar spectrum was kept and the other ones discarded. All the spectral processing was performed using MATLAB 2013a (Mathworks).

## 4. Conclusions

Applying hyphenated analytical tools to the crude extract obtained from the zoanthid *Parazoanthus axinellae* led to the identification of five new compounds, namely parazoanthines F–J. Although the presence of some of these compounds was proposed earlier [18], no chemical evidence of the structures had been given in the living organisms. The results obtained were demonstrated to be very useful for the identification of compounds in other organisms, especially protected or endangered species. This method demonstrated to be efficient for the identification of new compounds at a nanogram scale. More interestingly, the value of the results obtained by simulation and experiment in this study raises the question of an epistemological problem beyond the frame of this work. The creation of a model using the metabolome consistency concept (structure generation + spectra simulation) eventually demonstrated to be even more accurate than classical approach (extraction, purification and structure elucidation using 1D and 2D NMR) because not exposed to experimental risks (lack of accuracy and sensitivity). An increasing request of experiments is also an unbridgeable epistemic impediment in several scientific fields, although sometimes it can be bypass through an additional resource commitment (as in the present study). If resource saving is an important issue of modern science as chemists assert through the concept of beauty [19], the cost of psychological comfort of a systematic use of experience should not be underestimated.

An epistemology of the “why not”, *i.e.*, trusting the models, appears as a necessity for modern natural products chemistry anxious not to confine itself to a methodology based on experience, that despite its apparent seriousness, is a cumbersome relic [20].

## Supplementary Files

Supplementary File 1 Supporting Information (PDF, 1094 KB)
