# Supplementary material for: Metabolome Consistency: Additional Parazoanthines from the Mediterranean Zoanthid Parazoanthus Axinellae
Source: Metabolites. 2014 May 30;4(2):421–32. doi: 10.3390/metabo4020421 (PMC4101514; doi:10.3390/metabo4020421)
Supplement: Supplementary File 1 — Supporting Information (PDF, 1094 KB) [file metabolites-04-00421-s001.pdf]

Supporting Information

Figure S1	Proposed fragmentation mechanisms	S2
Figure S2	Experimental MS/MS spectrum of compound **6**	S3
Figure S3	Comparison between simulated and experimental MS/MS spectra of compound **6**	S3
Figure S4	Experimental MS/MS spectrum of compound **7**	S4
Figure S5	Comparison between simulated and experimental MS/MS spectra of compound **7**	S4
Figure S6	Experimental MS/MS spectrum of compound **8**	S5
Figure S7	Comparison between simulated and experimental MS/MS spectra of compound **8**	S5
Figure S8	Experimental MS/MS spectrum of compound **9**	S6
Figure S9	Comparison between simulated and experimental MS/MS spectra of compound **9**	S6
Figure S10	Experimental MS/MS spectrum of compound **10**	S7
Figure S11	Comparison between simulated and experimental MS/MS spectra of compound **10**	S7
Figure S12	Putative biogenetic links of parazoanthine compounds	S8
Table S1	List of generated compounds not found in the extract	S9

Figure S1. Proposed fragmentation mechanisms.

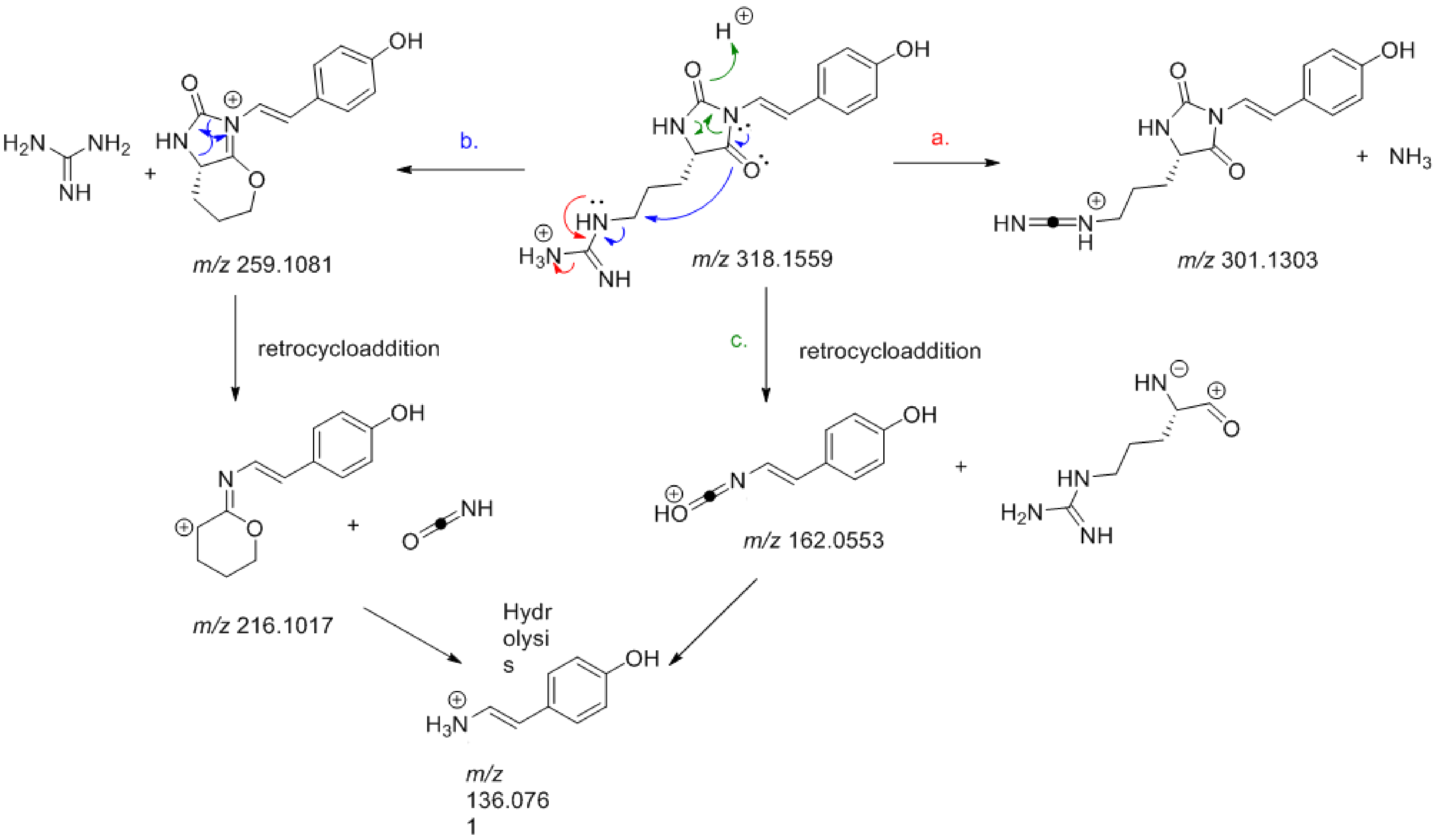

Figure S2. Experimental MS/MS spectrum of compound **6**.

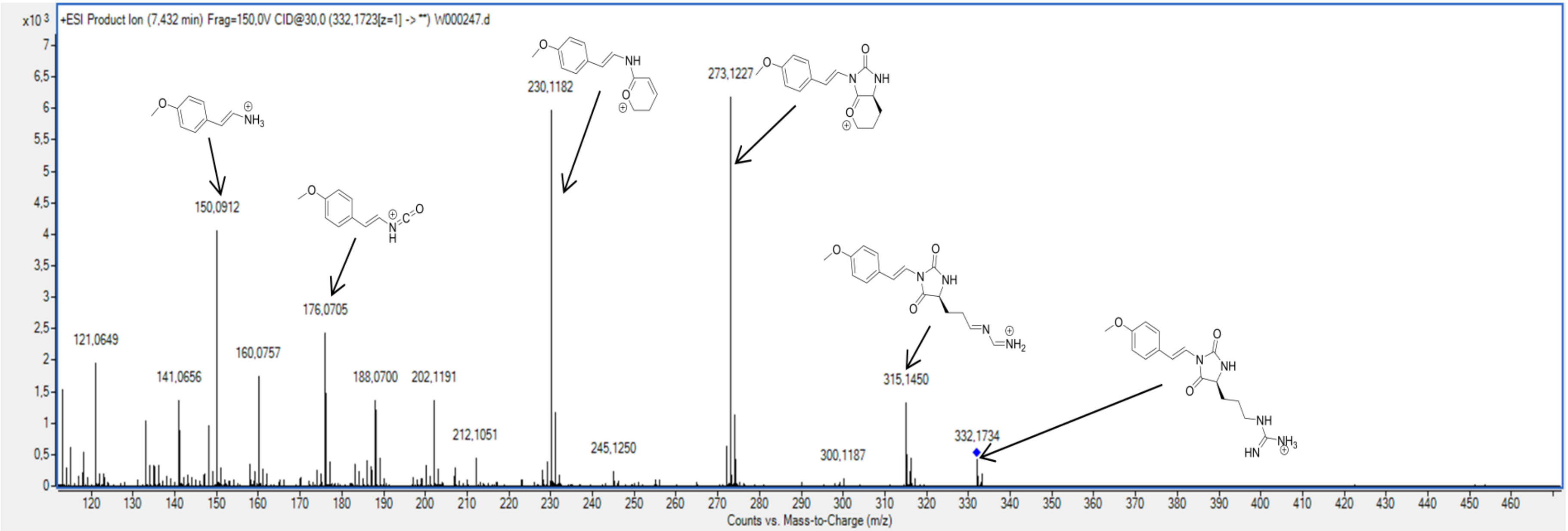

Figure S3. Comparison between simulated and experimental MS/MS spectra of compound **6**.

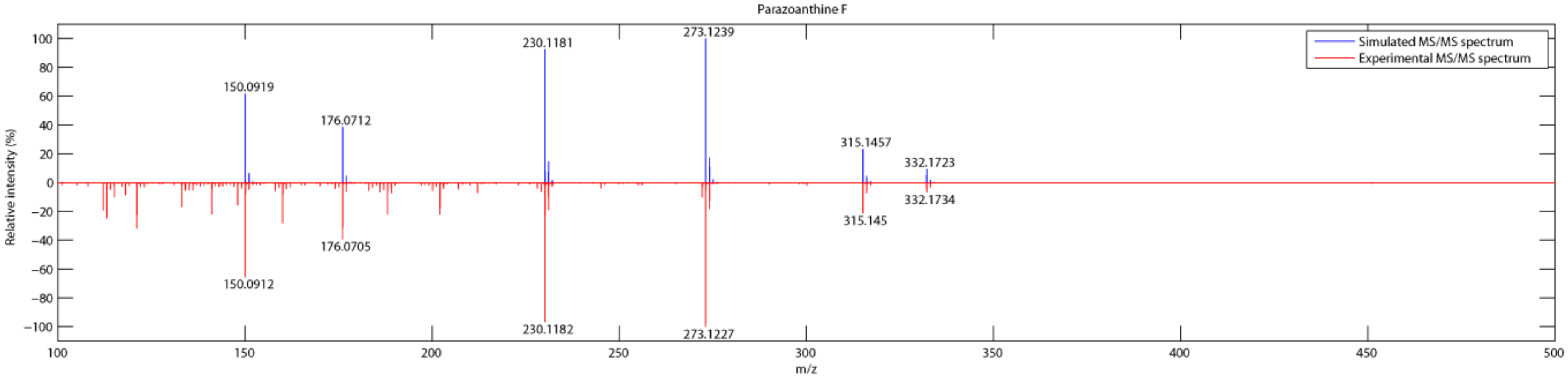

Figure S4. Experimental MS/MS spectrum of compound **7**.

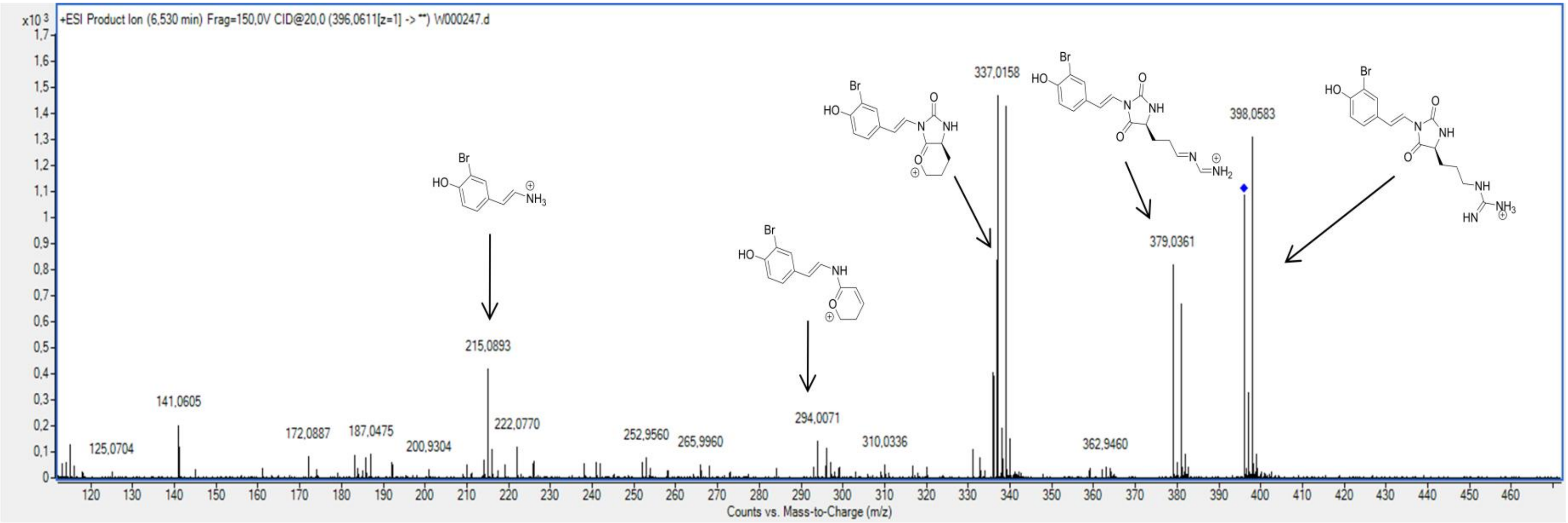

Figure S5. Comparison between simulated and experimental MS/MS spectra of compound **7**.

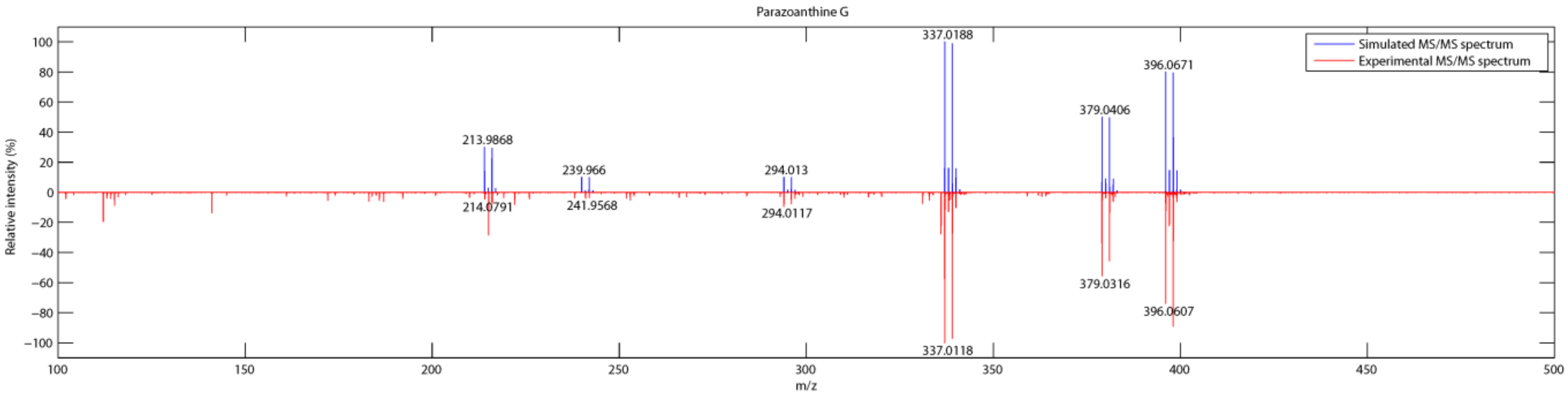

Figure S6. Experimental MS/MS spectrum of compound **8**.

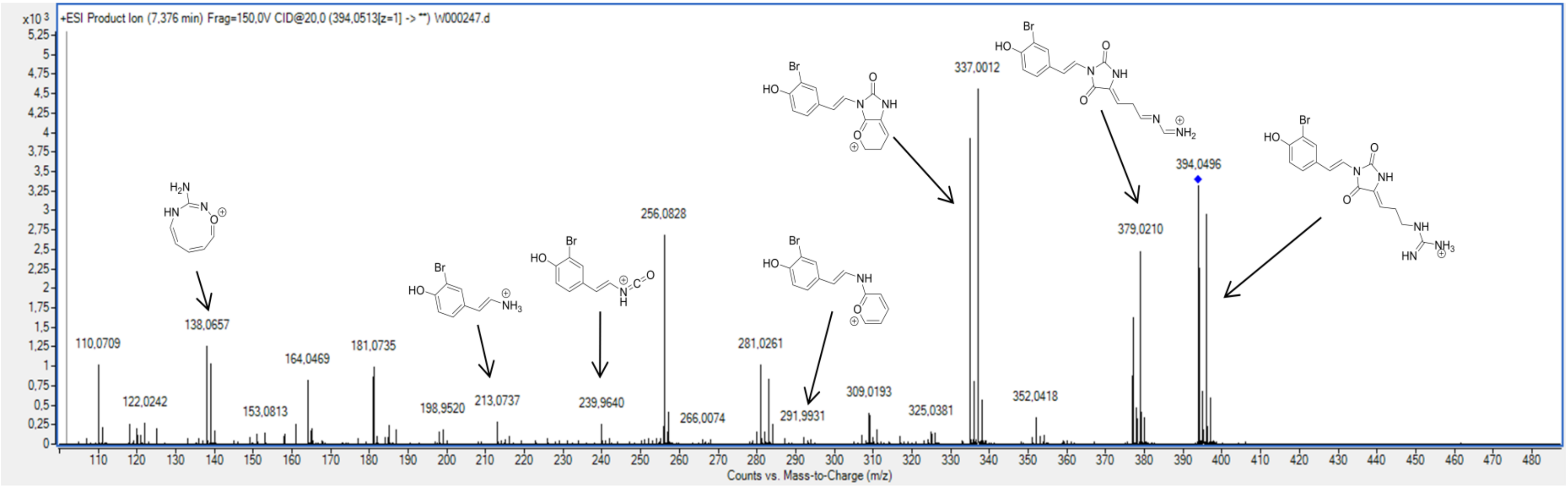

Figure S7. Comparison between simulated and experimental MS/MS spectra of compound **8**.

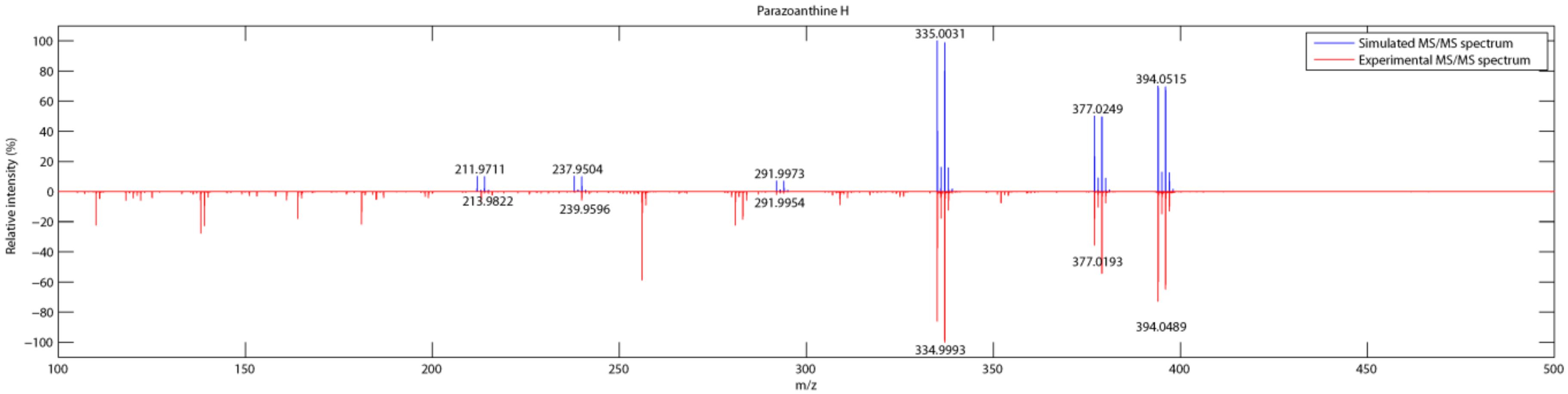

Figure S8. Experimental MS/MS spectrum of compound **9**.

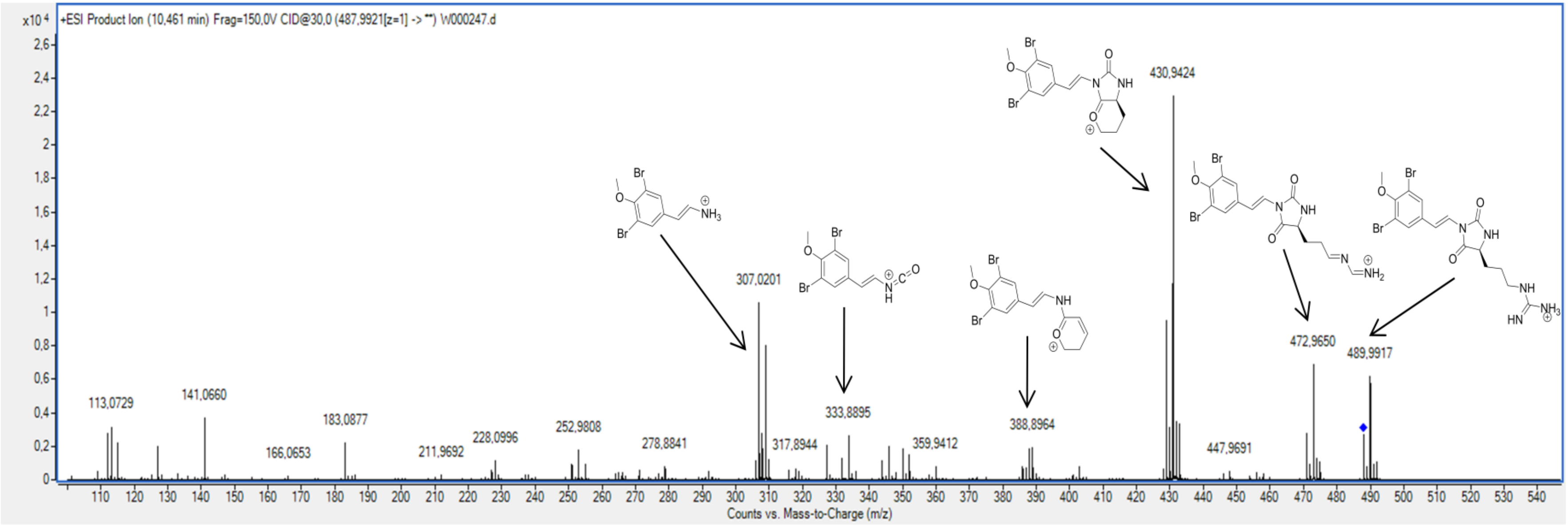

Figure S9. Comparison between simulated and experimental MS/MS spectra of compound **9**.

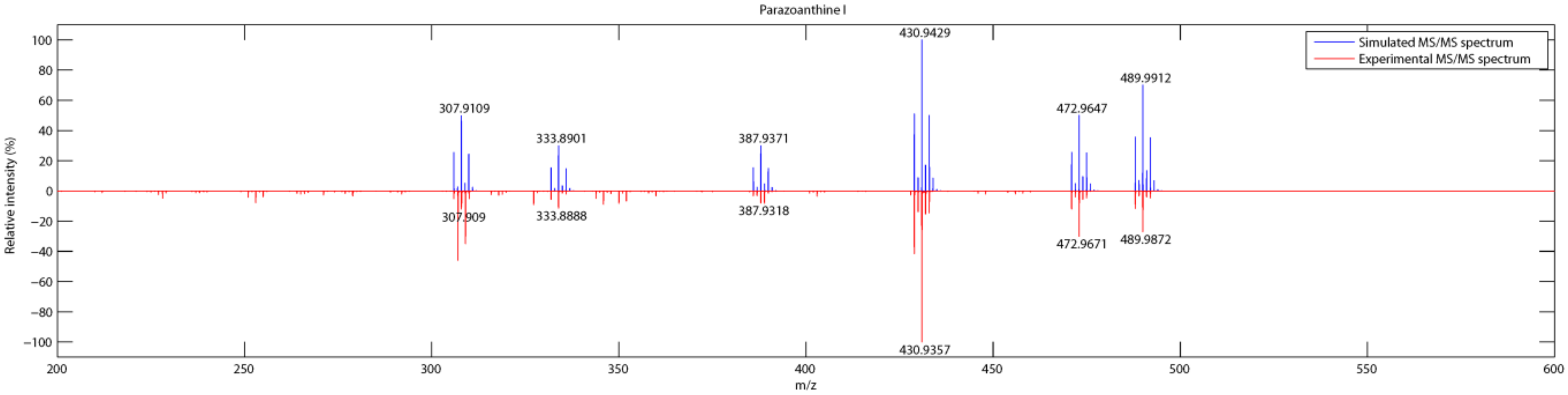

Figure S10. Experimental MS/MS spectrum of compound **10**.

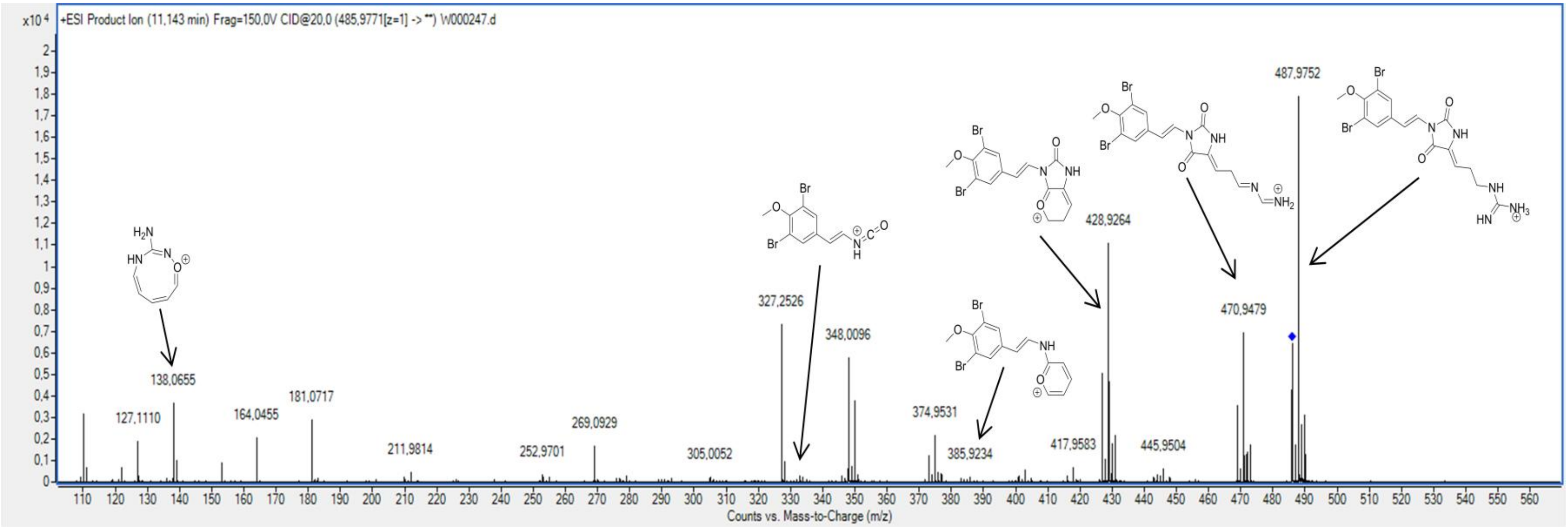

Figure S11. Comparison between simulated and experimental MS/MS spectra of compound **10**.

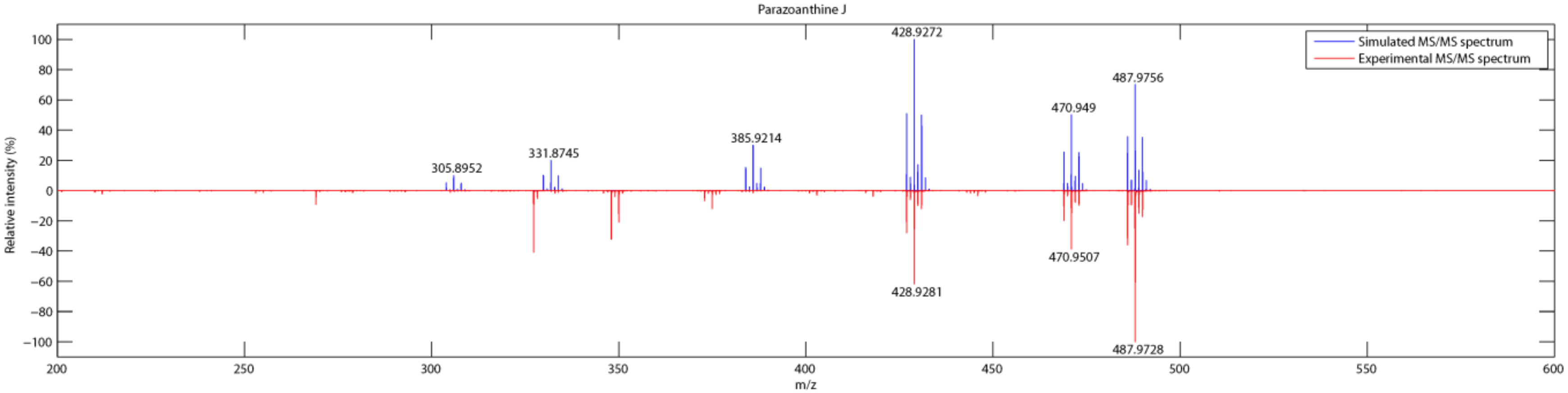

Figure S12. Putative biogenetic links of parazoanthine compounds.

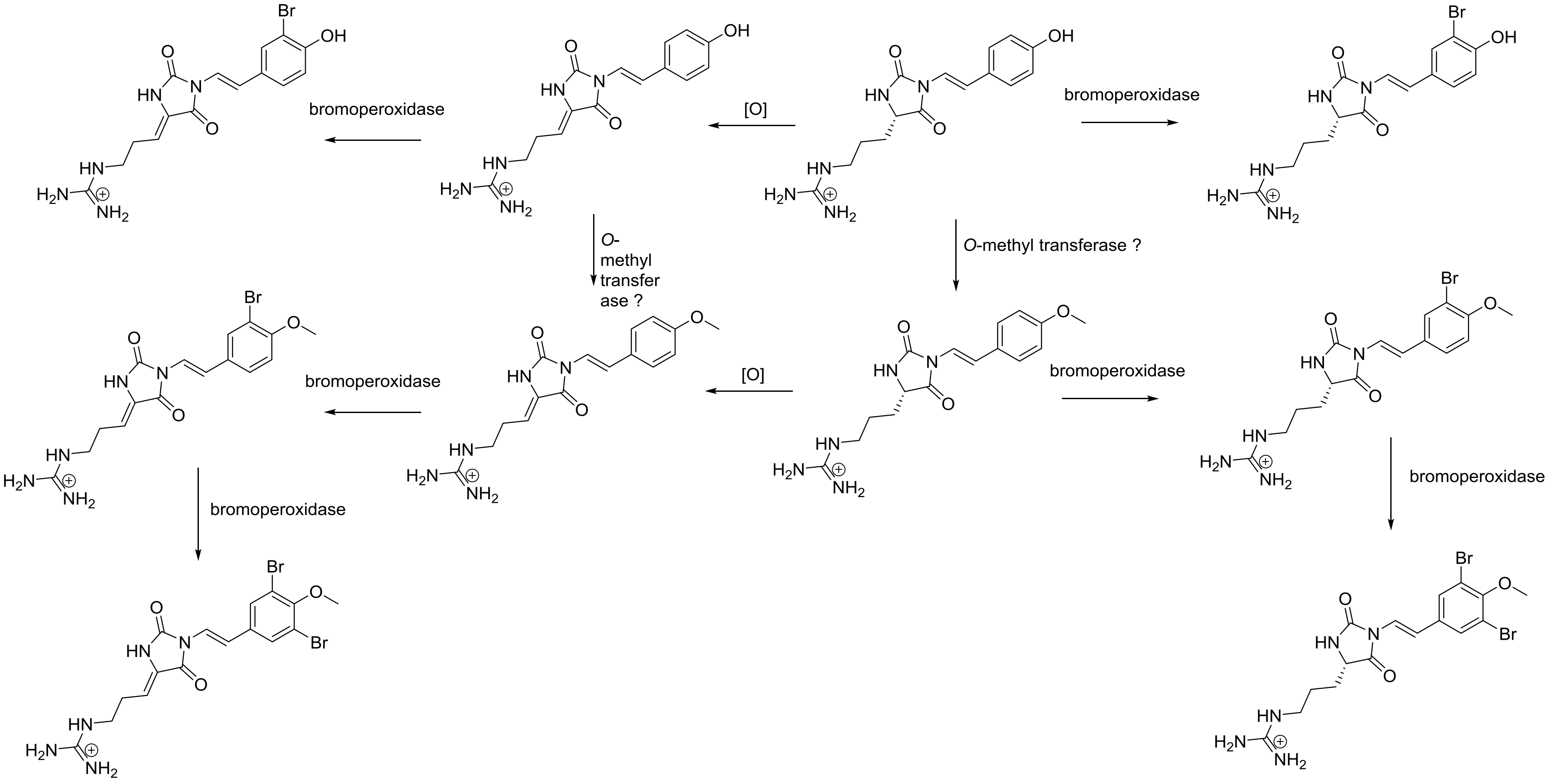

Table S1. List of generated compounds not found in the extract.

Empirical Formula	*m/z*					
	$[M+H]^+$	$[M-NH_3+H]^+$	$[M-CH_5N_3+H]^+$	$[M-C_2H_6N_4O+H]^+$	$[M-C_6H_{10}N_4O+H]^+$	$[M-C_7H_{10}N_4O_2+H]^+$
C16H20FN5O3	349.15502	332.12847	290.10667	247.10086	195.06956	167.07464
C17H22FN5O3	363.17067	346.14412	304.12232	261.11651	209.08521	181.09029
C18H24FN5O3	377.18632	360.15977	318.13797	275.13216	223.10086	195.10594
C15H17F2N5O3	353.12995	336.10340	294.08160	251.07579	180.04608	152.05117
C16H19F2N5O3	367.14560	350.11905	308.09725	265.09144	194.06173	166.06682
C17H21F2N5O3	381.16125	364.13470	322.11290	279.10709	208.07738	180.08247
C18H23F2N5O3	395.17690	378.15035	336.12855	293.12274	222.09303	194.09812
C16H20IN5O3	457.06108	440.03453	398.01274	355.00692	302.97562	274.98071
C17H22IN5O3	471.07673	454.05018	412.02839	369.02257	316.99127	288.99636
C18H24IN5O3	485.09238	468.06583	426.04404	383.03822	331.00692	303.01201
C15H17I2N5O3	568.94208	551.91553	509.89373	466.88791	287.95215	259.95723
C16H19I2N5O3	582.95773	565.93118	523.90938	480.90356	301.96780	273.97288
C17H21I2N5O3	596.97338	579.94683	537.92503	494.91921	315.98345	287.98853
C18H23I2N5O3	610.98903	593.96248	551.94068	508.93486	329.99910	302.00418
C16H20ClN5O3	341.10300	324.07645	324.06388	295.06114	243.02984	215.03492
C17H22ClN5O3	355.11865	338.09210	338.07953	309.07679	257.04549	229.05057
C18H24ClN5O3	369.13430	352.10775	352.09518	323.09244	271.06114	243.06622
C15H17Cl2N5O3	361.04838	344.02183	344.00925	315.00651	228.00636	200.01145
C16H19Cl2N5O3	375.06403	358.03748	358.02490	329.02216	242.02201	214.02710
C17H21Cl2N5O3	389.07968	372.05313	372.04055	343.03781	256.03766	228.04275
C18H23Cl2N5O3	403.09533	386.06878	386.05620	357.05346	270.05331	242.05840
C16H18FN5O3	347.13937	330.11282	288.09102	245.08521	193.05391	165.05899
C17H20FN5O3	361.15502	344.12847	302.10667	259.10086	207.06956	179.07464
C18H22FN5O3	375.17067	358.14412	316.12232	273.11651	221.08521	193.09029
C15H15F2N5O3	351.11430	334.08775	292.06595	249.06013	178.03043	150.03552
C16H17F2N5O3	365.12995	348.10340	306.08160	263.07579	192.04608	164.05117

Table S1. *Cont.*

Empirical Formula	***m/z***					
	**$[M+H]^+$**	**$[M-NH_3+H]^+$**	**$[M-CH_5N_3+H]^+$**	**$[M-C_2H_6N_4O+H]^+$**	**$[M-C_6H_{10}N_4O+H]^+$**	**$[M-C_7H_{10}N_4O_2+H]^+$**
C17H19F2N5O3	379.14560	362.11905	320.09725	277.09144	206.06173	178.06682
C18H21F2N5O3	393.16125	376.13470	334.11290	291.10709	220.07738	192.08247
C16H18IN5O3	455.04543	438.01888	395.99709	352.99127	300.95997	272.96506
C17H20IN5O3	469.06108	452.03453	410.01274	367.00692	314.97562	286.98071
C18H22IN5O3	483.07673	466.05018	424.02839	381.02257	328.99127	300.99636
C15H15I2N5O3	566.92643	549.89988	507.87808	464.87226	285.93650	257.94158
C16H17I2N5O3	580.94208	563.91553	521.89373	478.88791	299.95215	271.95723
C17H19I2N5O3	594.95773	577.93118	535.90938	492.90356	313.96780	285.97288
C18H21I2N5O3	608.97338	591.94683	549.92503	506.91921	327.98345	299.98853
C16H18ClN5O3	339.08735	322.06080	322.04823	293.04549	241.01419	213.01927
C17H20ClN5O3	353.10300	336.07645	336.06388	307.06114	255.02984	227.03492
C18H22ClN5O3	367.11865	350.09210	350.07953	321.07679	269.04549	241.05057
C15H15Cl2N5O3	359.03273	342.00618	341.99360	312.99086	225.99071	197.99580
C16H17Cl2N5O3	373.04838	356.02183	356.00925	327.00651	240.00636	212.01145
C17H19Cl2N5O3	387.06403	370.03748	370.02490	341.02216	254.02201	226.02710
C18H21Cl2N5O3	401.07968	384.05313	384.04055	355.03781	268.03766	240.04275
